# Rapid Authentication of the Herbal Medicine Plant Species *Aralia continentalis* Kitag. and *Angelica biserrata* C.Q. Yuan and R.H. Shan Using ITS2 Sequences and Multiplex-SCAR Markers

**DOI:** 10.3390/molecules21030270

**Published:** 2016-02-29

**Authors:** Wook Jin Kim, Byeong Cheol Moon, Sungyu Yang, Kyeong Suk Han, Goya Choi, A Yeong Lee

**Affiliations:** K-herb Research Center, Korea Institute of Oriental Medicine, Daejeon 305-811, Korea; ukgene@kiom.re.kr (W.J.K.); sgyang81@kiom.re.kr (S.Y.); hks86@kiom.re.kr (K.S.H.); serparas@kiom.re.kr (G.C.); lay7709@kiom.re.kr (AY.L.)

**Keywords:** *Aralia continentalis*, *Angelica biserrata*, Araliae Continentalis Radix, Angelicae Pubescentis Radix, internal transcribed spacer (ITS), sequence characterized amplified region (SCAR) marker, molecular authentication, multiplex-polymerase chain reaction (multiplex-PCR)

## Abstract

Accurate identification of the plant species that are present in herbal medicines is important for quality control. Although the dried roots of *Aralia continentalis* (Araliae Continentalis Radix) and *Angelica biserrata* (Angelicae Pubescentis Radix) are used in the same traditional medicine, namely *Dok-Hwal* in Korean and *Du-Huo* in Chinese, the medicines are described differently in the national pharmacopeia. Further confusion arises from the distribution of dried *Levisticum officinale* and *Heracleum moellendorffii* roots as the same medicine. Medicinal ingredients from all four plants are morphologically similar, and discrimination is difficult using conventional methods. Molecular identification methods offer rapidity and accuracy. The internal transcribed spacer 2 (ITS2) region of the nuclear ribosomal RNA gene (rDNA) was sequenced in all four plant species, and the sequences were used to design species-specific primers. Primers for each species were then combined to allow sample analysis in a single PCR reaction. Commercial herbal medicine samples were obtained from Korea and China and analyzed using the multiplex assay. The assay successfully identified authentic medicines and also identified inauthentic or adulterated samples. The multiplex assay will be a useful tool for identification of authentic Araliae Continentalis Radix and/or Angelicae Pubescentis Radix preparations in Korea and China.

## 1. Introduction

The botanical identity of herbal medicines can be controversial as a result of historical naming patterns. Similar names can be used for different medicinal plants (homonyms), and different names can be used for the same medicinal plants (synonyms). The roots of *Aralia continentalis* Kitagawa (Araliaceae) and *Angelica pubescens* Maxim. f. *biserrata* Shan et Yuan (a synonym of *An. biserrata* C.Q. Yuan and R.H. Shan) are described as the same herbal medicine. These two roots are used in Korean and Chinese traditional medicine to dispel wind and treat painful obstructions in the lower body [1]. In Korean traditional medicine, only the dried root of *Ar. continentalis* is described as Araliae Continentalis Radix, namely *Dok-Hwal*, in the Korean Pharmacopeia [2]. However, the root of *Angelica pubescens* Maxim. f. *biserrata* Shan et Yuan (a synonym of *Angelica biserrata* C.Q. Yuan and R.H Shan) is described as the same herbal medicine, namely *Du-Huo* in Chinese (Angelicae Pubescentis Radix), in the Chinese Pharmacopeia [1,3]. As a result of importation of *An. biserrata* from China, both *Ar. continentalis* and *An. biserrata* have been used as *Dok-Hwal* in Korea. Furthermore, roots of *Heracleum moellendorffii* Hance often have been used as Aralia Continentalis Radix because the roots of this plant have only slight morphological differences from those of *Ar. continentalis*. Finally, *Levisticum officinale* W.D.J. Koch roots are frequently found as adulterants in Angelicae Pubescentis Radix and Angelicae Sinensis Radix in Chinese herbal markets. Thus, the roots of four plant species (*Ar. continentalis*, *An. biserrata*, *H. moellendorffii*, and *L. officinale*) have all been used as the same herbal medicine [4,5]. The roots of the four species are morphologically similar, and precise species differentiation of sliced or processed roots is challenging when conventional authentication criteria such as shape, texture, or odor are used [6]. Reliable methods for the identification of *Ar. continentalis*, *An. biserrata*, *H. moellendorffii*, and *L. officinale* species in herbal preparations are therefore desirable [7].

Objective and reliable identification of plant materials can be achieved using molecular genetic tools such as polymerase chain reaction (PCR) and sequencing. DNA barcoding sequences are often used for species identification and evaluation of phylogenetic relationships. DNA barcoding uses short nuclear or chloroplast DNA sequences that contain conserved regions to facilitate PCR amplification and divergent regions to allow species differentiation [7,8,9,10,11]. Two chloroplast gene sequences (*mat*K and *rbc*L) and the internal transcribed spacer (ITS) sequences of nuclear ribosomal RNA (rDNA) are widely used for DNA barcoding in plants [4,11,12,13,14,15,16,17,18,19]. The ITS2 region, which is part of the rDNA ITS region, has been used extensively for authentication of medicinal plants. The ITS2 region has a high rate of nucleotide substitution, and sequences are highly specific in plants. Although the ITS2 region is a standard barcoding region, cumbersome amplification, sequencing, and alignment processes have hampered its utility for identification of individual species [13,20]. Sequence characterized amplified region (SCAR) markers can be designed based on the amplified regions and used for rapid, simple, cheap, and reproducible species identification. SCAR markers have been used for successful authentication of a range of medicinal plants and herbal medicines [9,21,22,23,24,25].

In this study, ITS2 sequences from *Ar. continentalis*, *An. biserrata*, *H. moellendorffii*, and *L. officinale* were used to design species-specific primers and SCAR markers for the identification of individual species. Multiplex-SCAR markers were developed for concurrent identification of the four medicinal plants, and the multiplex assay was used to examine herbal medicines distributed as *Dok-Hwal*, Araliae Continentalis Radix, and Angelicae Pubescentis Radix in Korean and Chinese herbal markets. This assay will be valuable for assessing the integrity of Araliae Continentalis Radix and/or Angelicae Pubescentis Radix and for identifying adulterated medicines.

## 2. Results

### 2.1. Analysis of Sequences and Phylogenetic Relationships

**Figure 1:**
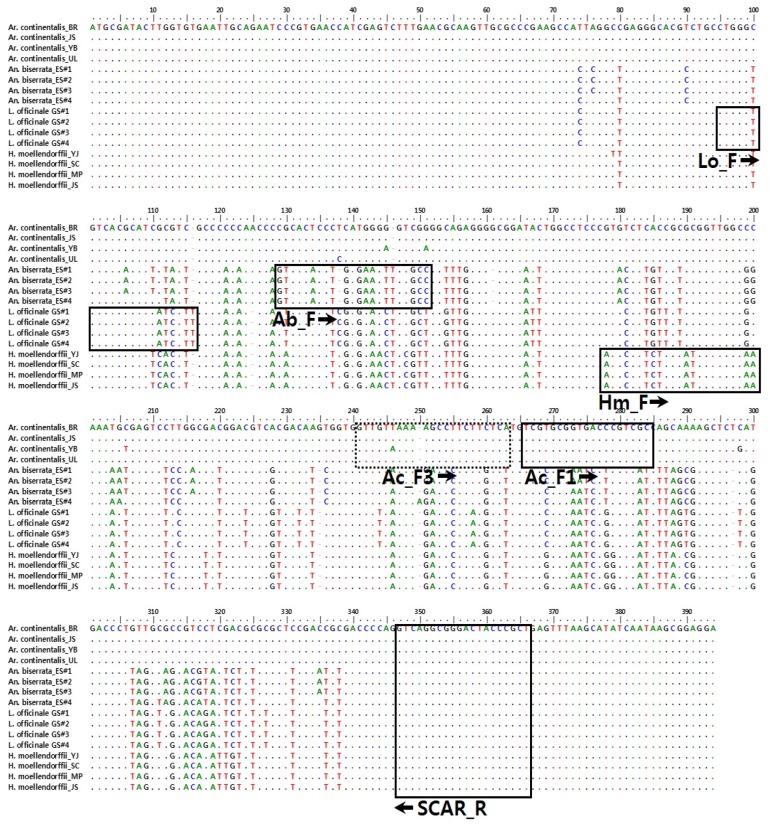
Comparative analysis of ITS2 sequences and design of species-specific SCAR primers. Boxes indicate SCAR primer sequences used in this study. Primer names are indicated under the boxes.

ITS2 DNA fragments of the expected length (~400 bp) were successfully amplified from 16 plant samples (four each of *Ar. continentalis*, *An. biserrata*, *H. moellendorffii*, and *L. officinale* as shown in Table 1). Amplicons were cloned into the pGEM-T Easy vector and then sequenced using T7 and SP6 universal vector primers. ITS2 sequences for each sample were deposited in GenBank (see Materials and Methods for accession numbers). ITS2 sequence lengths were 391 bp in *Ar. continentalis*, 388 bp in *An. biserrata*, 390 bp in *L. officinale*, and 389 bp in *H. moellendorffii* (Table 2). Sequences were aligned to a length of 394 bp. Inter-specific sequence variability was 0.0000% − 0.0063% ± 0.0115% and intra-specific sequence variability was 0.1519% ± 0.0811% – 0.2634% ± 0.0104% (Table 2 and Supplementary Table S1). Among the four species, 0–3 indel regions and 10–51 nucleotide substitutions specific to the species level were identified (Table 2). These indels and nucleotide substitutions allowed marker nucleotides to be identified for each species (Table 3 and Figure 1). Species-specific marker nucleotides were identified at 54 positions (including three species-specific indels) for *Ar. continentalis*, 14 positions (including two species-specific indels) for *An. biserrata*, 10 positions for *L. officinale*, and 15 positions for *H. moellendorffii* (Table 2 and Table 3). These species-specific markers could be used to identify authentic Araliae Continentalis Radix and Angelicae Pubescentis Radix.

To evaluate the phylogenetic relationships among the four species, phylogenetic trees were constructed by applying the neighbor-joining (NJ) method to the entire sequences of ITS2. All 16 individual samples were clustered into four groups, constituting monophyletic clades within each species that reflected the intra-specific variation (Supplementary Figure S1). As shown by the phylogenetic tree, *An. biserrata* was genetically closer to *L. officinale* than to the other two species, while *Ar. continentalis* was more distant from the other three species. Bootstrap values clustered at the species level ranged from 90% to 100% (Supplementary Figure S1). To distinguish species based on the phylogenetic tree, the minimum bootstrap value was 90% in ITS2 barcode sequences. From this phylogenetic analysis, we confirmed that identification of the four herbaceous species can be achieved using sequence variability in ITS2 sequences.

### 2.2. Development of Species-Specific SCAR Markers

**Figure 2:**
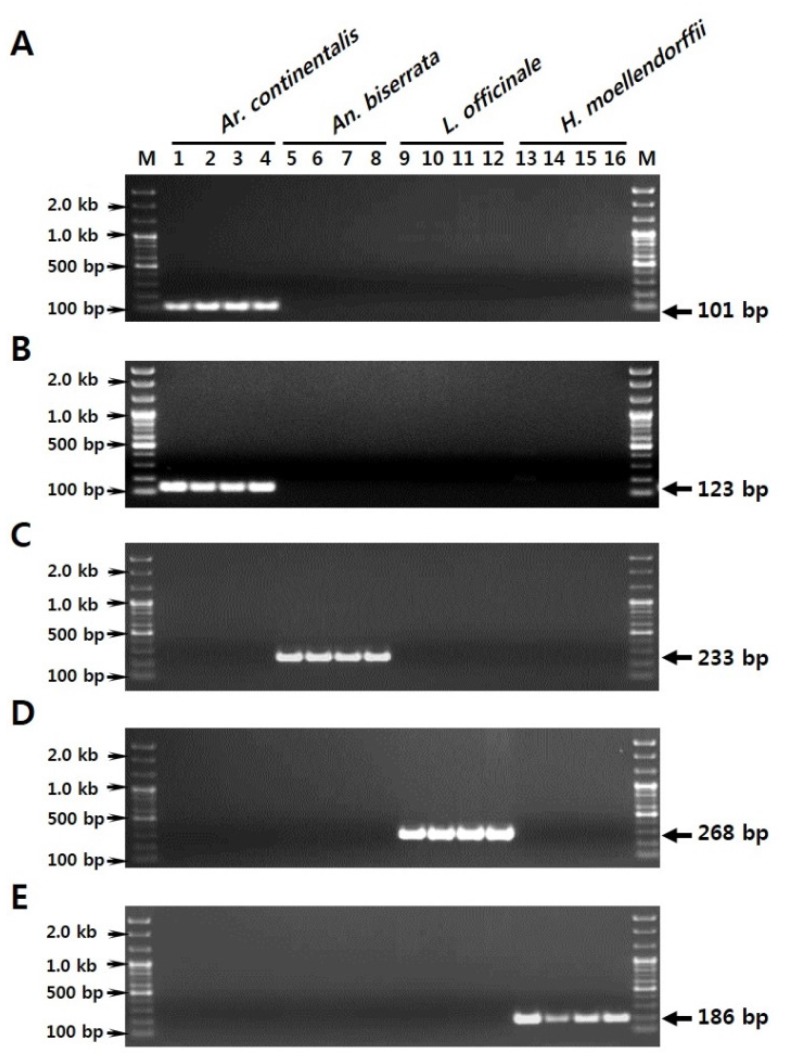
Development of species-specific SCAR markers from sequence variations in the ITS2 region. (**A**) PCR amplification of an *Ar. continentalis*–specific SCAR marker using Ac_F1 and SCAR_R primers; (**B**) PCR amplification of an *Ar. continentalis*–specific SCAR marker using Ac_F3 and SCAR_R primers; (**C**) PCR amplification of an *An. biserrata*–specific SCAR marker using Ab_F and SCAR_R primers; (**D**) PCR amplification of an *L. officinale*–specific SCAR marker using Lo_F and SCAR_R primers; (**E**) PCR amplification of a *H. moellendorffii*–specific SCAR marker using Hm_F and SCAR_R primers. Primer sequences are given in Table 4. Lanes 1–16 correspond to those listed in Table 1. M represents a 100 bp DNA ladder. Arrowheads to the right and left of the panels indicate the precise sizes of the PCR products and DNA ladder, respectively.

Candidate regions for the design of species-specific primers (SCAR primers) were identified by comparative analysis of the ITS2 sequences from all 16 plant samples. Several forward SCAR primers were designed against sequence-variable regions. A common reverse primer, SCAR_R, was used. Primer specificities were tested with the 16 plant samples (Figure 1). The five SCAR primers amplified DNA fragments only in the target species (Table 4 and Figure 2), and amplicons were all of the expected sizes. Characteristic 101 and 123 bp amplicons were produced in *Ar. continentalis* with the Ac_F1 and Ac_F3 primers, respectively. Ac_F1 and Ac_F3 produced no amplicons with *An. biserrata*, *L. officinale*, or *H. moellendorffii* (Figure 2A,B). The Ac_F1 and Ac_F3 primers were therefore suitable for distinguishing *Ar. continentalis* from the other species. Similarly, the Ab_F, Lo_F, and Hm_F primers amplified unique PCR products only in *An. biserrata* (233 bp amplicon), *L. officinale* (268 bp amplicon), and *H. moellendorffii* (186 bp amplicon), respectively (Figure 2C–E). The five SCAR markers were therefore successful in distinguishing *Ar. continentalis*, *An. biserrata*, *L. officinale*, and *H. moellendorffii* at the DNA level.

### 2.3. Development of a Multiplex-SCAR Method for Authentication of Herbal Medicine

**Figure 3:**
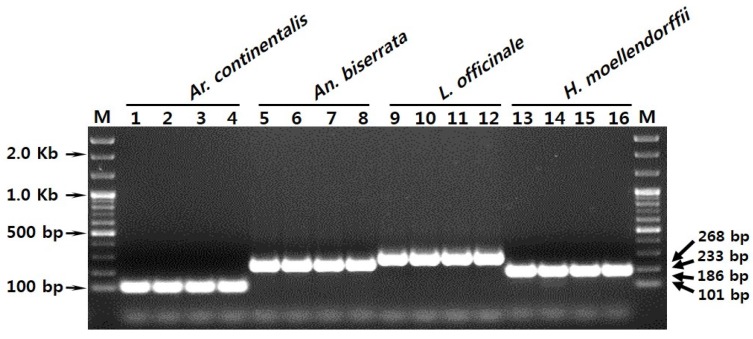
Development of a multiplex-SCAR assay using a combination of species-specific primers and multiplex-PCR. Amplicons were produced using primers Ac_F1, Ab_F, Lo_F, Hm_F, and SCAR_R in a single PCR reaction. M represents a 100 bp DNA ladder. Lanes 1–16 correspond to those listed in Table 1. Arrowheads to the right and left of the panels indicate the precise sizes of the PCR products and DNA ladder, respectively.

SCAR primers were used to develop a multiplex-PCR assay for distinguishing Araliae Continentalis Radix and Angelicae Pubescentis Radix and for the identification of *Ar. continentalis*, *An. biserrata*, *L. officinale*, and *H. moellendorffii*. Amplicon size was used to identify species. The primer combination that consisted of Ac_F1, Ab_F, Lo_F, Hm_F, and SCAR_R was assessed for combined use in single PCR reactions. Amplification with this primer combination yielded four distinct DNA fragments that corresponded to the amplicons produced by the individual SCAR reactions (Figure 2A,C–E and Figure 3). Primers obtained in this study were appropriate for use in a multiplex-SCAR assay for identifying the four herbaceous plant species.

**Figure 4:**
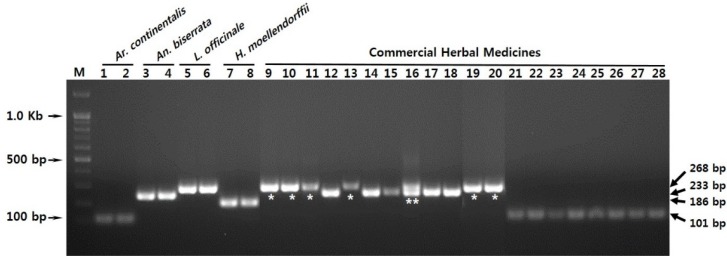
Analysis of commercial herbal medicine samples using the multiplex-SCAR assay. Lanes 1–8: control plant materials; Lanes 9–28: commercial herbal medicines collected from Chinese and Korean herbal markets. Sample details are provided in Supplementary Table S2. M represents a 100 bp DNA ladder. Lanes 1–28 correspond to those listed in Supplementary Table S2. Arrowheads to the right and left of the panels indicate the precise sizes of the PCR products and DNA ladder, respectively. * and ** represent the inauthentic and mixed medicinal samples against the product labels, respectively.

The multiplex-SCAR assay method was used to test 20 commercial herbal medicine samples. Ten samples of Angelicae Pubescentis Radix were purchased from Chinese herbal markets and 10 samples of Araliae Continentalis Radix were purchased from Korean herbal markets (Supplementary Table S2). Of the 20 commercial samples, seven were inauthentic: six samples contained only dried *L. officinale* root (Figure 4, lanes 9, 10, 11, 13, 19, and 20) and one sample was a mixture of *An. biserrata* and *L. officinale* (Figure 4, lane 16). These results demonstrated the utility of the multiple-SCAR assay for use in plant materials and processed herbal medicines. The results also indicated that *L. officinale* roots were frequently substituted for Araliae Continentalis Radix and Angelicae Pubescentis Radix in the herbal market. The multiplex-SCAR assay method developed in this study will be useful for rapid and effective detection of adulterated medicines and for discrimination of authentic Araliae Continentalis Radix and Angelicae Pubescentis Radix from inauthentic substitutes.

## 3. Discussion

The accurate identification of plant species in herbal medicines is critical for quality control. Inexpensive alternative plants, in particular *L. officinale*, are frequently substituted in Angelicae Pubescentis Radix (Chinese herbal name, *Du-Huo*). Similarly, *H. moellendorffii* is frequently found in Araliae Continentalis Radix (Korean herbal name, *Dok-Hwal*) in Korea [3,5]. Although *L. officinale* is of the *Levisticum* genus, the morphological features and dried root slices are very similar in appearance to those of *An. biserrata* and *A. sinensis*, which are in the *Angelica* genus. Yuan *et al.* (2015) considered *L. officinale* to be a member of the *Angelica* genus, according to DNA barcode–based molecular phylogenic analysis [5]. Our comparative analysis of ITS2 sequences supports this: inter-species variability between *L. officinale* and *An. biserrata* was the lowest of the four different species (Table 2). The phylogenetic analysis also supported the suggestion that *L. biserrata* could be considered a member of the *Angelica* genus (Supplementary Figure S1). Confusion regarding taxonomic features and the distributions of herbal preparations led Araliae Continentalis Radix and Angelicae Pubescentis Radix, in Korea and China, respectively, to be considered the same medicine, and also allowed contamination of medicines with *L. officinale* and *H. moellendorffii* roots. A rapid, reliable technique to distinguish the two herbal medicines from one another and identify adulterants is therefore of value in both Korea and China. Here, we developed a multiplex molecular authentication method and used this to confirm that *L. officinale* is the main adulterant in both Korea and China (Figure 4 and Supplementary Table S2).

Molecular markers such as random amplified polymorphic DNAs (RAPD), amplified fragment length polymorphisms (AFLP), DNA barcodes with short sequences, super DNA barcodes with complete plastid genome sequences, SCARs, and multiplex-SCARs have been used widely for species identification and quality control [7,8,10,22,26,27]. The use of SCAR markers for species identification is simple, reliable, and reproducible, and multiplexing simplifies reactions and improves diagnostic power [13]. SCAR markers, including multiplex-SCAR markers, are often developed from polymorphic RAPD amplicons obtained from genomic profiling [22,23,24,25]. In this study, ITS2 DNA barcode sequences were used to identify variable regions suitable for use as SCAR markers [28]. Inter-species comparisons of ITS2 regions from the 16 plant samples revealed sufficient sequence variability to allow identification of *Ar. continentalis*, *An. biserrata*, *L. officinale*, and *H. moellendorffii* at the species level (Table 3). ITS sequences are frequently used for the identification of medicinal plants. However, subcloning, sequencing, and comparative analysis are required [8,9,10,13]. To simplify the procedure, we used the species-specific sequence regions to design SCAR primers that would amplify unique DNA fragments in each of the four plant species. Furthermore, amplicon lengths were sufficiently different in the different species to allow multiplexing (Figure 1). The SCAR primers recognized only the correct species, and PCR products were amplified successfully in both the SCAR and multiplex-SCAR reactions (Figure 2 and Figure 3). The multiplex-SCAR assay method established in this study was used to test samples of commercially available herbal medicines in Korea and China (Figure 4). The resulting sequences of SCAR amplicons amplified from the 20 commercial herbal medicine samples also revealed species identical to those in the multiplex-SCAR assays. The rapid multiplex-SCAR assay developed in this study avoids many of the problems associated with DNA barcoding, such as the need for a database, large-scale analyses, and analysis of complex datasets, and will be valuable in the standardization and authentication of Araliae Continentalis Radix and Angelicae Pubescentis Radix.

## 4. Materials and Methods

### 4.1. Plant and Herbal Medicine Materials

Four samples each of *Ar. continentalis*, *An. biserrata*, *H. moellendorffii*, and *L. officinale* were used in the analysis (Table 1). Samples were collected from different native habitats and from cultivated farms in Korea and China. Samples were frozen in liquid nitrogen and then stored at −70 °C. Herbal medicines were purchased from herbal markets in different geographical regions. All plant materials and herbal medicines were assigned accession numbers, and specimens were preserved in the Korean Herbarium of Standard Herbal Resources (herbarium code KIOM) at the Korea Institute of Oriental Medicine (KIOM). Species identification was performed by the Classification and Identification Committee of the KIOM, which comprises nine experts in the fields of plant taxonomy, botany, pharmacognosy, and herbology.

### 4.2. Preparation of Genomic DNA

Genomic DNA was extracted from frozen leaves and herbal medicines using a DNeasy^®^ Plant Mini Kit (Qiagen, Valencia, CA, USA) according to the manufacturer’s protocol. Purity and concentration of DNA were assessed using a spectrophotometer (Nanodrop ND-1000, Nanodrop, Wilmington, DE, USA) and 1.5% agarose gel electrophoresis. The final DNA concentration used for PCR amplification was approximately 20 ng/μL in TE buffer. Extracted DNA samples were stored at −20 °C.

### 4.3. PCR Amplification of ITS2

ITS2 regions were amplified using ITS2-s2f (5′-ATG CGA TAC TTG GTG TGA AT-3′) and ITS4 (5′-TCC TCC GCT TAT TGA TAT GC-3′) primers, using previously described amplification parameters [29,30]. PCR amplifications were performed in 50 μL reaction volumes containing 10 mM Tris-HCl (pH 9.0), 2.5 mM MgCl_2_, 200 μM each dNTP, 10 mM (NH_4_)_2_SO_4_, 0.5 U *Taq* DNA polymerase (Solgent, Daejeon, Korea), 0.6 μM each primer, and approximately 15 ng of template DNA. PCR amplification was performed using a DNA Engine Dyad^®^ PTC-0220 (Bio-Rad, Foster City, CA, USA). The parameters were as follows: 95 °C for 5 min; 35 cycles of 30 s at 95 °C, 30 s at 55 °C, and 2 min at 72 °C; and a final extension for 5 min at 72 °C. PCR products were separated using 1.5% agarose gel electrophoresis with a 100 bp DNA ladder (Solgent, Daejeon, Korea).

### 4.4. Nucleotide Sequence and Phylogenetic Analysis and Development of SCAR Markers

Amplified ITS2 DNA fragments were extracted from agarose gels using a Gel Extraction Kit (Qiagen, Valencia, CA, USA) and subcloned into the pGEM-T Easy vector (Promega, Madison, WI, USA). Inserted fragments were sequenced in both directions using an automatic DNA sequence analyzer (ABI 3730, Applied Biosystems Inc., Foster City, CA, USA). ITS2 sequences from the four samples of each species were deposited in NCBI GenBank with the following accession numbers: *Ar. continentalis*, KT944663–KT944666; *An. biserrata*, KT944668–KT944670; *L. officinale*, KT944671–KT944674; and *H. moellendorffii*, KT944675–KT944678.

Approximately 400-bp ITS2 sequences were assembled and edited using BioEdit version 7.2.5 [31]. The contigs were aligned to analyze the intra- and inter-species variations in the sequences. For the analysis of sequence identity and evolution, inter- or intra-species genetic distances were calculated using the Kimura-2-parameter (K2P) model in MEGA 6.0 software. The phylogenetic analysis based on the entire ITS2 sequences was performed by MEGA version 6.06 [32,33]. The phylogenetic tree was constructed using the NJ method with the K2P model, pairwise deletion for gaps/missing data treatment, and 1000 replications for bootstrapping with *Mycosphaerella nyssicola* (KJ504767) as an outgroup control.

Species-specific primers were designed for amplification of SCAR regions. The 16 ITS2 sequences were aligned using the ClustalW package within BioEdit software (biological sequence editing software, Bioedit, version 7.2.5 [31]), and several candidate regions were identified with species-specific indels and nucleotide substitutions (Figure 1 and Table 4). PCR reactions were performed to confirm primer specificity. Reactions were performed in 30 μL and contained the same basic components as for ITS2 region amplification alongside 0.5 μM of the species-specific primers. Amplification conditions were as follows: pre-denaturation at 95 °C for 5 min; 35 cycles of 95 °C for 1 min, 63 °C for 30 s, and 72 °C for 2 min; and a final extension at 72 °C for 5 min. Amplified fragments were verified using 1.5% agarose gel electrophoresis.

### 4.5. Development of the Multiplex-SCAR Assay

For multiplex-SCAR analysis, four species-specific forward primers and one common reverse primer were combined in a single PCR reaction. Optimal amplification conditions were determined by altering the following parameters: annealing time (20–60 s), annealing temperature (57 °C–65 °C), number of PCR cycles (23–40 cycles), primer concentrations (0.25, 0.5, 0.75, and 1.0 μM of each primer), and primer combinations (Ac_F1 or Ac_F3). To verify the accuracy and specificity of the multiplex-SCAR assay, multiplex-PCR reactions were performed using total genomic DNA from individual plant species and from commercially available herbal medicines. PCR products were analyzed with 1.5% agarose gel electrophoresis.

## 5. Conclusions

Species-specific features of ITS2 sequences in *Ar. continentalis*, *An. biserrata*, *L. officinale*, and *H. moellendorffii* were identified through comparative analysis and used to develop a multiplex-SCAR assay for species-level identification. The assay was used to authenticate commercially available samples of the herbal medicines Araliae Continentalis Radix and Angelicae Pubescentis Radix, and to identify adulterated samples. The simple, rapid multiplex-SCAR assay will be useful in the authentication of Araliae Continentalis Radix and Angelicae Pubescentis Radix preparations and will help to identify adulterated products.

## Figures and Tables

**Table 1:** Plant materials used in this study.

Name		Habitat Information	Voucher Number	Lane in Gel
Scientific Name	Herbal Name			
*Aralia continentalis* Kitag. = *Aralia cordata* var. *continentalis* (Kigag.) Y.C. Chu	Araliae Continentalis Radix	Jusan, Boryeong, Chungnam, Korea	KIOM201301006224	1
		Janggye, Jangsu, Jeonbuk, Korea	KIOM201201004852	2
		Antu County, Jilin province, China	KIOM201201005561	3
		Ulleung, Gyeongbuk, Korea	KIOM2013KR05-36	4
*Angelica biserrata* C.Q. Yuan & R.H. Shan = *Angelica pubescens* f. *biserrata* R.H. Shan & C.Q. Yuan	Angelicae Pubescentis Radix	Badong, Enshi, Hubei, China	KIOM200801001319	5
		Badong, Enshi, Hubei, China	KIOM200801001320	6
		Badong, Enshi, Hubei, China	KIOM200801001321	7
		Badong, Enshi, Hubei, China	KIOM200801001483	8
*Levisticum officinale* W.D.J. Koch	-^1^	Lanzhou, Gansu, China	KIOM2011CN02-19	9
		Lanzhou, Gansu, China	KIOM2011CN02-20	10
		Lanzhou, Gansu, China	KIOM2011CN02-21	11
		Lanzhou, Gansu, China	KIOM2011CN02-22	12
*Heracleum moellendorffii* Hance	-^1^	Punggi Yeongju, Gyeongbuk, Korea	KIOM201101003889	13
		Seolcheon, Muju, Jeonbuk, Korea	KIOM200901002079	14
		Mupung, Muju, Jeonbuk, Korea	KIOM200801001576	15
		Jeoksang, Muju, Jeonbuk, Korea	KIOM200801001227	16

^1^ No official herbal name.

**Table 2:** Characteristics of ITS2 barcode sequences.

Species	Constant Length (bp)	Aligned Length (bp)	Intra-Species Variability	Inter-Species Variability	Species-Specific Marker Nucleotide	
					Indels	Substitutions
***Ar. continentalis***	391	394	0.0000 ± 0.0000	0.2634 ± 0.0104	3	51
***An. biserrata***	388	394	0.0063 ± 0.0115	0.1586 ± 0.0830	2	12
***L. officinale***	390	394	0.0000 ± 0.0000	0.1519 ± 0.0811	0	10
***H. moellendorffii***	389	394	0.0034 ± 0.0013	0.1550 ± 0.0693	0	15

**Table 3:** Summary of species-specific indels and nucleotide substitutions in the ITS2 DNA barcode region of *Ar. continentalis* and closely related medicinal plant species.

Nucleotide Position	80	90	100	111	112	113	115	116	121	123	128	129	130	134	137	138	139	141	142	143	144	145	146	148	149	150
*Ar. continentalis*	C	T	C	G	C	G	C	-	C	C	C	C	G	T	C	T	C	T	G	G	G	G	-	T	C	G
*An. biserrata*	T	C	T	·	T	A	T	·	A	A	A	G	T	A	T	-	G	G	A	A	G	T	T	·	G	C
*L. officinale*	T	·	T	A	T	C	T	T	A	A	A	·	T	·	T	C	G	G	·	A	G	C	T	·	G	C
*H. moellendorffii*	T	·	T	C	A	C	T	·	A	A	A	·	A	·	T	T	G	G	·	A	A	C	T	C	G	T
Nucleotide position	151	154	155	156	157	161	166	167	168	178	180	181	184	185	186	187	190	191	199	200	204	205	212	218	220	224
*Ar. continentalis*	G	C	A	G	A	G	T	A	C	G	G	T	C	A	C	C	G	C	C	C	T	G	C	G	C	C
*An. biserrata*	C	T	T	T	G	-	A	·	T	·	A	C	T	G	T	·	·	·	G	G	A	A	T	·	T	·
*L. officinale*	T	G	T	T	G	·	A	T	T	·	·	C	T	G	T	T	·	·	G	·	A	·	T	·	T	T
*H. moellendorffii*	T	T	T	T	G	·	A	·	T	A	·	C	T	C	T	·	A	T	A	A	A	·	T	T	T	·
Nucleotide position	228	232	234	235	236	244	246	251	252	255	258	260	263	269	273	274	275	276	278	279	283	284	286	287	288	
*Ar. continentalis*	A	C	A	G	T	G	T	A	G	T	T	C	A	T	G	T	G	A	C	C	G	C	A	G	C	
*An. biserrata*	G	·	T	-	C	·	A	G	A	C	·	G	T	C	A	A	T	C	T	·	A	T	T	T	A	
*L. officinale*	G	T	T	-	·	T	A	G	A	C	A	G	T	C	A	A	T	C	G	·	A	T	T	T	A	
*H. moellendorffii*	G	·	T	-	·	·	A	G	A	C	·	G	T	G	A	A	T	C	G	G	A	T	T	T	A	

Dots (·) indicate nucleotides identical to those of *Ar. continentalis*; Dashes (-) represent nucleotide deletions at the aligned nucleotide positions; Bold characters and dashes (-) represent species-specific substitutions and indels, respectively.

**Table 4:** Sequences of SCAR primers and the specificity of amplified DNA fragments and their sizes.

Primer Direction	Primer Name	Primer Sequence (5′ → 3′)	Specificity (Species)	Amplicon Size (bp)
Forward	Ac_F1	TCG TGC GGT GAC CCG TCG C	*Ar. continentalis*	101 bp
	Ac_F3	GTT GTT AAA AGC CTT CTT CTC A	*Ar. continentalis*	123 bp
	Ab_F	GTC ACA CCT GAG AAG TTG TGC C	*An. biserrata*	233 bp
	Lo_F	CTG GGT GTC ACG CAT CAT CTT T	*L. officinale*	268 bp
	Hm_F	ATG CCT TCT CGC ATG GTT GGC AA	*H. moellendorffii*	186 bp
Reverse	SCAR_R	AGC GGG TAG TCC CGC CTG AC	All four species	

## Supplementary Materials

The following are available online at www.mdpi.com/link/1420-3049/21/3/270/s1: Figure S1: Phylogenetic relationship of four medicinal plants based on the ITS2 sequences, Table S1: Sequence divergences of ITS2 DNA barcode regions among *Ar. continentalis* and closely related medicinal plants species, Table S2: List of plant materials and commercial herbal medicines used to confirm the multiplex-SCAR assay.
