# Supplementary material for: Rapid Authentication of the Herbal Medicine Plant Species Aralia continentalis Kitag. and Angelica biserrata C.Q. Yuan and R.H. Shan Using ITS2 Sequences and Multiplex-SCAR Markers
Source: Molecules. 2016 Feb 29;21(3):270. doi: 10.3390/molecules21030270 (PMC6273786; doi:10.3390/molecules21030270)
Supplement: Supplementary file 1 [file molecules-21-00270-s001.pdf]

Supplementary Materials: Rapid Authentication of the Herbal Medicine Plant Species *Aralia continentalis* Kitag. and *Angelica biserrata* C.Q. Yuan & R.H. Shan Using ITS2 Sequences and Multiplex-SCAR Markers

Wook Jin Kim, Byeong Cheol Moon, Sungyu Yang, Kyeong Suk Han, Goya Choi and A Yeong Lee

Table S1. Sequence divergences of ITS2 DNA barcode regions among the *Ar. continentalis* and closely related medicinal plants species.

	1	2	3	4	5	6	7	8	9	10	11	12	13	14	15	16
1	-															
2	0.0000	-														
3	0.0000	0.0000	-													
4	0.0000	0.0000	0.0000	-												
5	0.2784	0.2784	0.2784	0.2784	-											
6	0.2784	0.2784	0.2784	0.2784	0.0000	-										
7	0.2784	0.2784	0.2784	0.2784	0.0000	0.0000	-									
8	0.2593	0.2593	0.2593	0.2593	0.0210	0.0210	0.0210	-								
9	0.2650	0.2650	0.2650	0.2650	0.0942	0.0942	0.0942	0.0765	-							
10	0.2650	0.2650	0.2650	0.2650	0.0942	0.0942	0.0942	0.0765	0.0000	-						
11	0.2650	0.2650	0.2650	0.2650	0.0942	0.0942	0.0942	0.0765	0.0000	0.0000	-					
12	0.2650	0.2650	0.2650	0.2650	0.0942	0.0942	0.0942	0.0765	0.0000	0.0000	0.0000	-				
13	0.2535	0.2535	0.2535	0.2535	0.1154	0.1154	0.1154	0.1095	0.1024	0.1024	0.1024	0.1024	-			
14	0.2535	0.2535	0.2535	0.2535	0.1154	0.1154	0.1154	0.1095	0.1024	0.1024	0.1024	0.1024	0.0000	-		
15	0.2497	0.2497	0.2497	0.2497	0.1123	0.1123	0.1123	0.1064	0.0994	0.0994	0.0994	0.0994	0.0026	0.0026	-	
16	0.2497	0.2497	0.2497	0.2497	0.1123	0.1123	0.1123	0.1064	0.0994	0.0994	0.0994	0.0994	0.0026	0.0026	0.0000	-

Number 1–16 correspond to those listed in Table 1 (lane in gel).

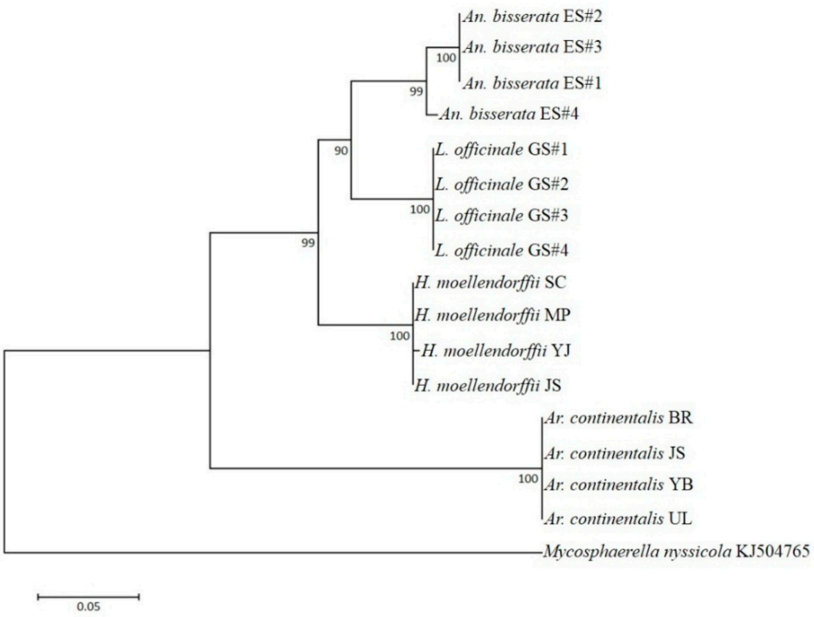

Figure S1. Phylogenetic tree with 1,000 bootstrap replicates based on ITS2 sequences of *Ar. contienentalis*, *An. biserrata*, *L. officinale*, and *H. moellendorffii* with an out-group, *Mycosphaerella nyssicola*. NJ bootstrap values greater than 60% are shown below the relevant branches.

Table S2. List of plant materials and commercial herbal medicines used to confirm the multiplex-SCAR assay.

Sample Name	Purchased Place	Origin of Samples	Collection Date	Lane in Gel
Ar. continentalis	Korea	Boryeong, Chungnam, Korea	2013. 08. 08	1
		Jangsu, Jeonbuk, Korea	2012. 09. 05	2
An. biserrata	China	Enshi, Hubei, China	2008. 08. 15	3
		Enshi, Hubei, China	2008. 08. 15	4
L. officinale	China	Lanzhou, Gansu, China	2011. 06. 21	5
		Lanzhou, Gansu, China	2011. 06. 21	6
H. moellendorffii	Korea	Yeongju, Gyeongbuk, Korea	2011. 09. 22	7
		Muju, Jeonbuk, Korea	2009. 08. 28	8
Angelicae Pubescentis Radix	China	Herbei, China	2015. 04. 20	9
		Gansu, China	2015. 04. 20	10
		Northeast, China	2015. 04. 20	11
		Hunan, China	2015. 04. 20	12
		Northeast, China	2015. 04. 20	13
		Sichuan, China	2015. 04. 20	14
		North Korea	2015. 04. 20	15
		Hubei, China	2015. 04. 20	16
		Guangxi, China	2015. 04. 20	17
		Anhui, Chia	2015. 04. 20	18
Araliae Continentalis Radix	Korea	Kyeongdong market, Seoul, Korea	2014. 06. 04	19
		Kyeongdong market, Seoul, Korea	2014. 06. 04	20
		Imsil, Jeonbuk, Korea	2014. 01. 29	21
		Jangsu, Jeonbuk, Korea	2014. 01. 29	22
		Damyang, Jeonnam, Korea	2014. 08. 14	23
		Damyang, Jeonnam, Korea	2014. 08. 14	24
		Jecheon, Chungbuk, Korea	2015. 02. 01	25
		Kyeongdong, Seoul, Korea	2015. 02. 01	26
		Jecheon, Chungbuk, Korea	2014. 06. 04	27
		Imsil, Jeonbuk, Korea	2014. 02. 14	28
